# Does noise shift or delete spikes?

**DOI:** 10.1186/1471-2202-14-S1-P246

**Published:** 2013-07-08

**Authors:** Sergej Voronenko, Benjamin Lindner

**Affiliations:** 1 Department of Physics, Humboldt-Universität zu Berlin, Berlin, 12489, Germany; 2 Bernstein Center for Computational Neuroscience, Berlin, 10115, Germany

## 

Stochastic leaky integrate-and-fire neurons are widely used to study properties of neural networks (e.g. [1]) as well as the spontaneous activity [2] and signal transmission [3] of single neurons. They have also been employed in studies of the 'common-noise' problem, i.e. the question of how correlated input to two cells causes output spike train correlations of these neurons [4].

In the cortex, input correlations seem to be weak [5] (in terms of the above sketch, c<<1), however, in the sensory periphery with a strong time-dependent stimulus the situation can be completely different (e.g.[6]). The two neurons receive the same strong stimulus (often modeled as a random signal) and each neuron is subject to a small amount of intrinsic noise (1-c<<1). Without the intrinsic noise two identical neurons would fire in complete synchrony. How does the weak noise change the spikes?

To address this question, we first study two versions of inhomogeneous Poisson processes. In one version the intrinsic noise can shift spike times, in the other the noise leads to deletions and additions of spikes. We construct these processes in such a way that the correlations between the modulated spike trains and the perturbing noise is the same in both versions. In this setup, it is possible to analytically calculate the cross-correlation between two spike trains with independent intrinsic noise. By comparison with extensive simulations of stochastic integrate-and-fire neurons we then inspect, which of the two modulation methods is more appropriate to capture the effect of a weak noise on the spike train of a dynamic neuron model.

**Figure 1:**
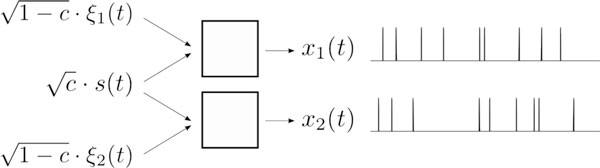
**Two neurons receive a common signal and distinct intrinsic noise**. c is the correlation parameter that tunes the strength of the input correlation.
